# Supplementary material for: Nanoparticles with curcumin and piperine modulate steroid biosynthesis in prostate cancer
Source: Sci Rep. 2025 Apr 19;15:13613. doi: 10.1038/s41598-025-98102-z (PMC12009323; doi:10.1038/s41598-025-98102-z)
Supplement: Supplementary file 1 — Supplementary Information. [file 41598_2025_98102_MOESM1_ESM.pdf]

Supplementary Material

Nanoparticles with Curcumin and Piperine Modulate Steroid Biosynthesis in Prostate Cancer

Jibira Yakubu [a,b,c], Evangelos Natsaridis[d], Therina du Toit[b,e], Isabel Sousa Barata [a,b,c], Oya Tagit[d], Amit V. Pandey [a,b,*]

[a] Paediatric Endocrinology, Diabetology and Metabolism, University Children's Hospital, Inselspital, Bern, Switzerland.
[b] Translational Hormone Research Program, Department of Biomedical Research, Faculty of Medicine, University of Bern, Bern, Switzerland.
[c] Graduate School for Cellular and Biomedical Sciences, University of Bern, Bern, Switzerland.
[d] Biointerfaces, Institute of Chemistry and Bioanalytics, University of Life Sciences FHNW, Muttenz, Switzerland.
[e] Department of Nephrology and Hypertension, University Hospital Bern, Inselspital, Bern, Switzerland.

*Address for Correspondence:

Prof. Dr. Amit V Pandey
Pediatric Endocrinology, University Children's Hospital Bern
Freiburgstrasse 15, KIKL C837, 3010 Bern, Switzerland
Email: amit.pandey@unibe.ch
Tel: 0041 31 632 9637

Supplementary Table S1. The cell viability results of the drugs on non-prostate cancer cell lines.

Cell viability results of the drugs on HEK293T cells										
	24 hours					48 hours				
**Drugs**	**%Control**			**Mean**	**SD**	**%Control**			**Mean**	**SD**
**DMSO**	99.9153	100.501	99.5841	100	0.46413	93.3076	98.3343	108.358	100	7.66223
**Polymer [Empty nanoparticle]**	100.332	100.107	99.561	100	0.39637	92.4785	105.185	102.336	100	6.66775
**Ethanol**	101.265	98.337	100.398	100	1.50397	109.019	88.603	102.378	100	10.4136
**Abiraterone**	133.215	130.787	130.716	131.573	1.42229	121.044	129.033	121.796	123.958	4.41148
**Abiraterone nanoparticle**	111.307	111.743	112.057	111.703	0.37671	86.0048	86.8621	85.889	86.2519	0.53157
**Curcumin**	74.646	73.3742	72.8	73.6067	0.94468	33.4154	38.3812	40.7866	37.5277	3.75896
**Curcumin nanoparticle**	87.1068	88.5939	87.0433	87.5813	0.87751	98.7113	86.1354	86.3333	90.3933	7.20427
**Curcumin_piperine nanoparticle**	83.067	83.4948	81.5627	82.7082	1.01478	98.7357	78.2272	69.8108	82.2579	14.8777
**Piperine**	84.6663	81.4508	85.5756	83.8976	2.16719	108.929	94.4789	121.999	108.469	13.7656
**Piperine nanoparticle**	122.264	122.985	123.227	122.825	0.50116	89.969	90.9575	105.059	95.3285	8.44131

Supplementary Figure S1: The calibration curve of Piperine

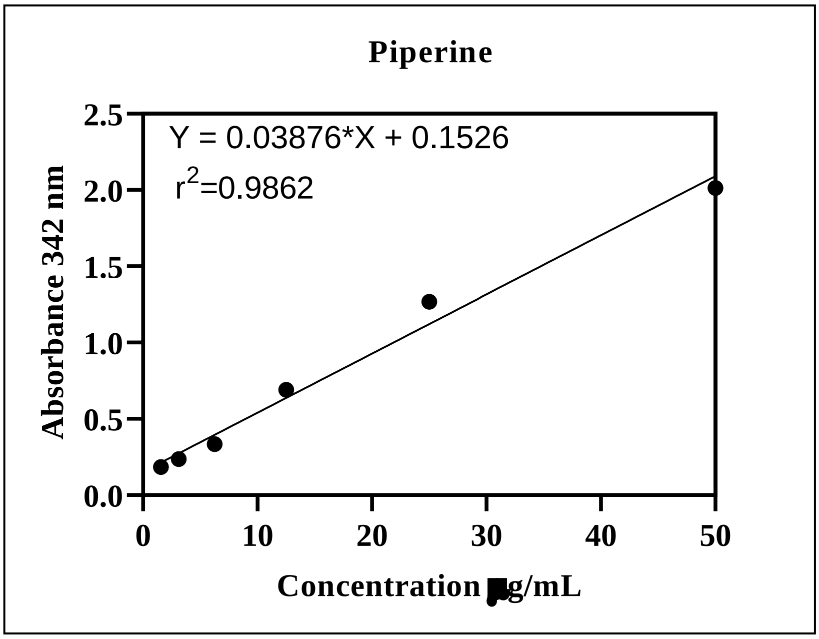

Supplementary Figure S2.1: Images from the fluorescence microscopy

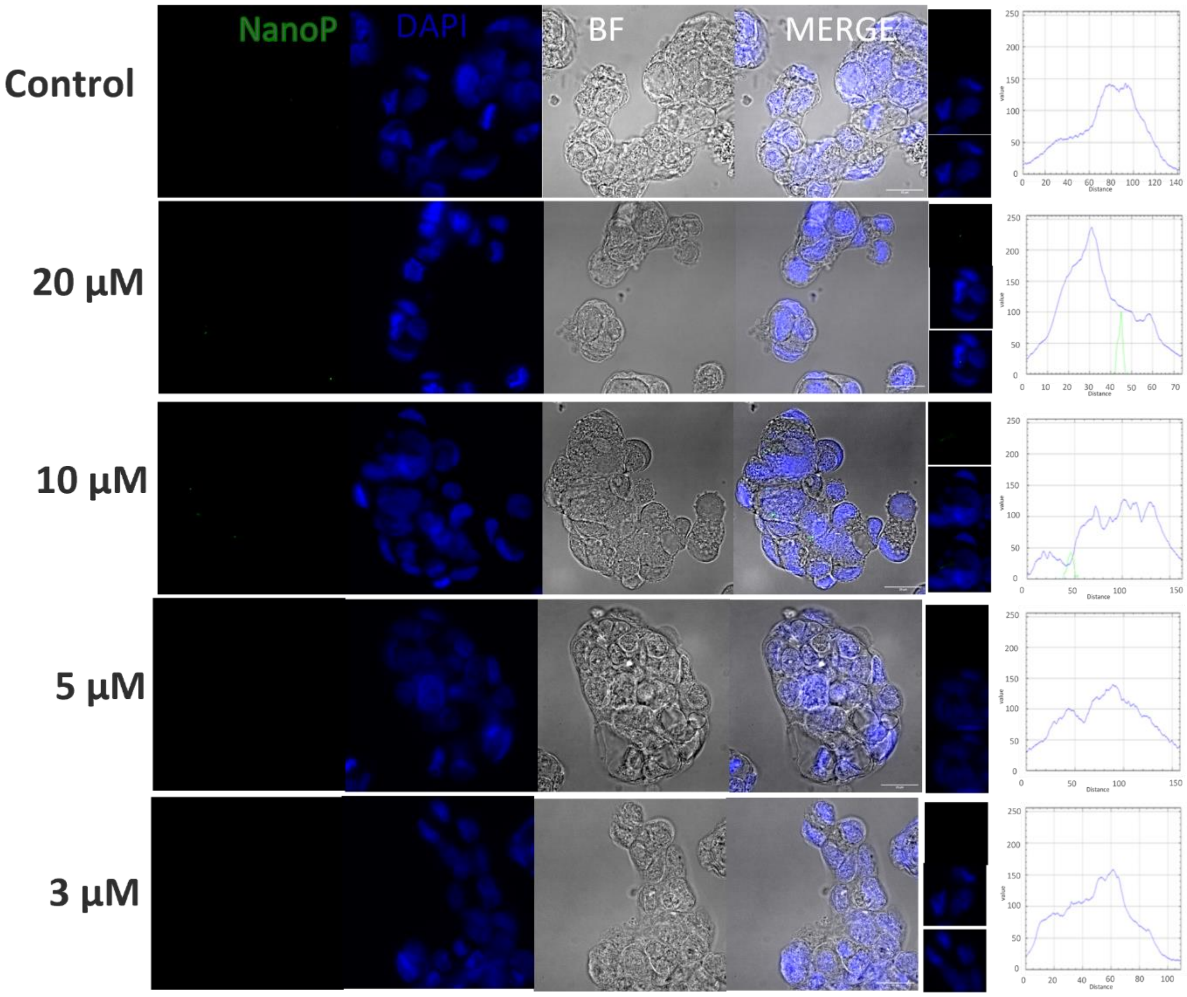

Supplementary Figure S2.1. Fluorescence microscopy of Curcumin in Caco-2 cells. Caco-2 cells were treated with increasing concentrations of Curcumin (20, 10, 5, 3, and 1 µM) for 4 hours. Cells were visualized using fluorescence microscopy to observe curcumin's cytosolic staining. **Scale bar**: 20 µm.

Supplementary Figure S2.2: Images from the fluorescence microscopy

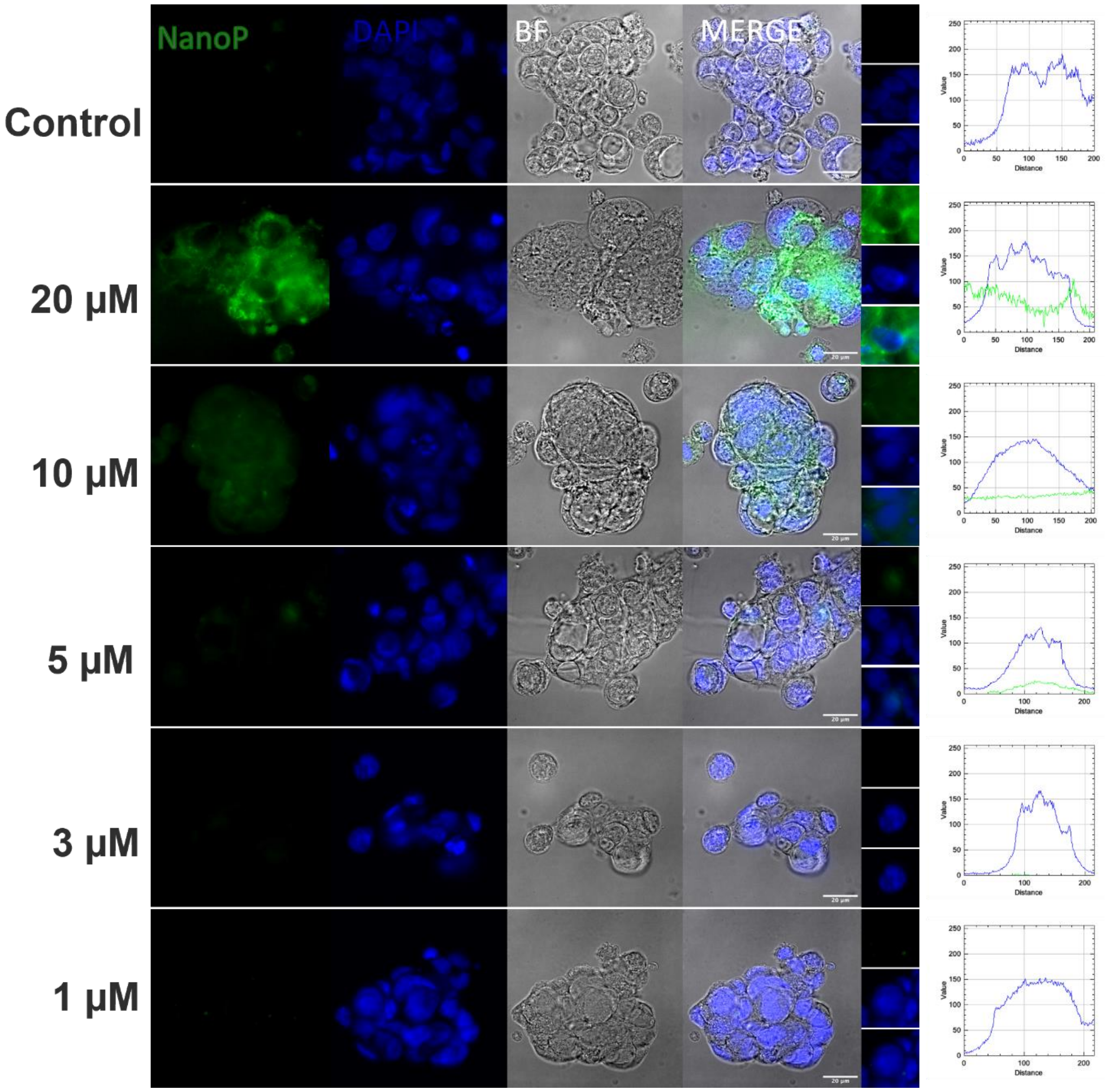

Supplementary Figure S2.2. Fluorescence microscopy of Curcumin nanoparticle in Caco-2 cells. Caco-2 cells were treated with increasing concentrations of Curcumin in the curcumin nanoparticle (20, 10, 5, 3, and 1 µM) for 4 hours. Cells were visualized using fluorescence microscopy to observe curcumin's cytosolic staining. **Scale bar**: 20 µm.

Supplementary Figure S2.3: Images from the fluorescence microscopy

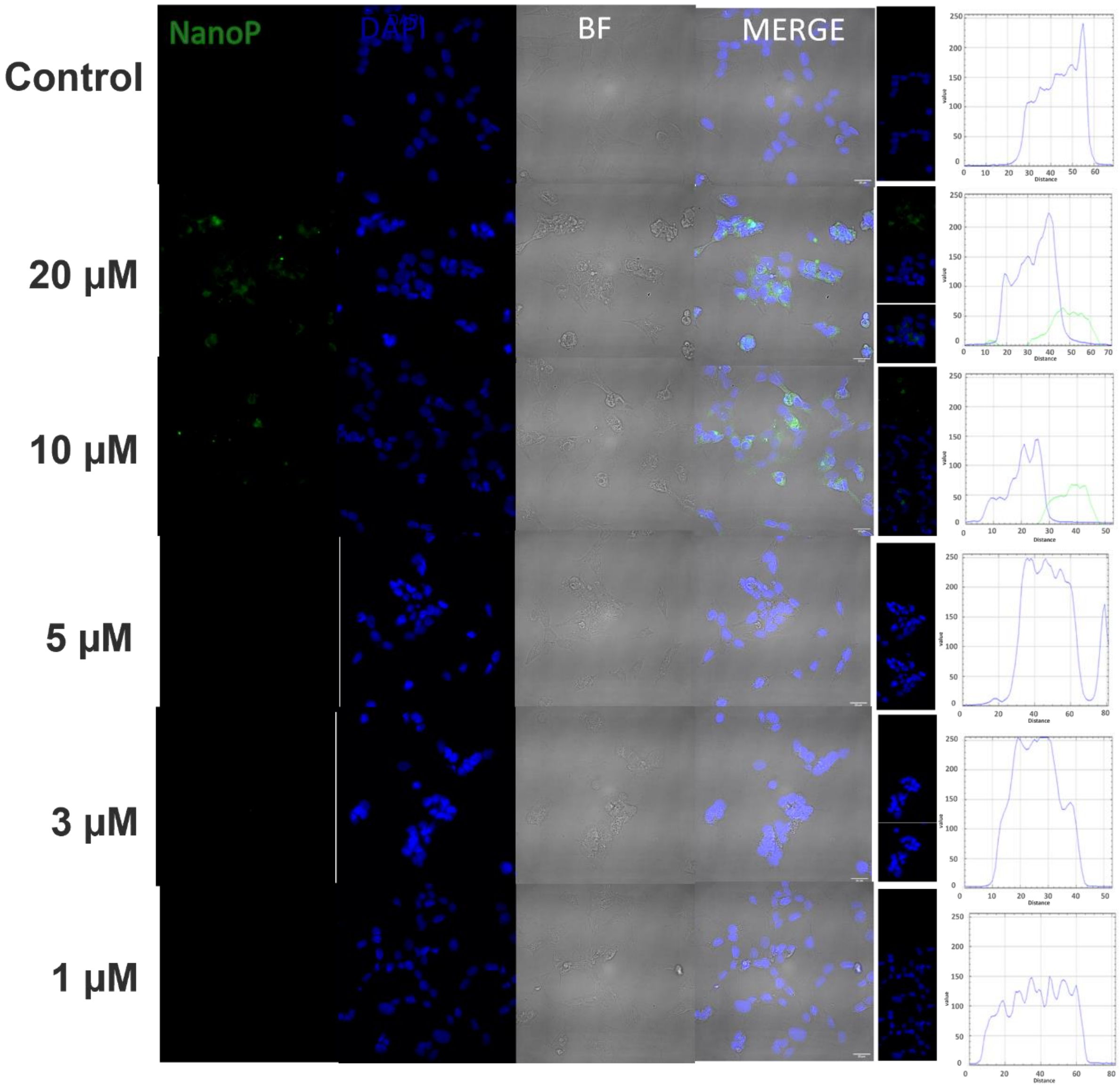

Supplementary Figure S2.3. Fluorescence microscopy of Curcumin in NCI-H295R cells. NCI-H295R cells were treated with increasing concentrations of Curcumin (20, 10, 5, 3, and 1 μM) for 4 hours. Cells were visualized using fluorescence microscopy to observe curcumin's cytosolic staining. **Scale bar**: 20 μm.

Supplementary Figure S2.4: Images from the fluorescence microscopy

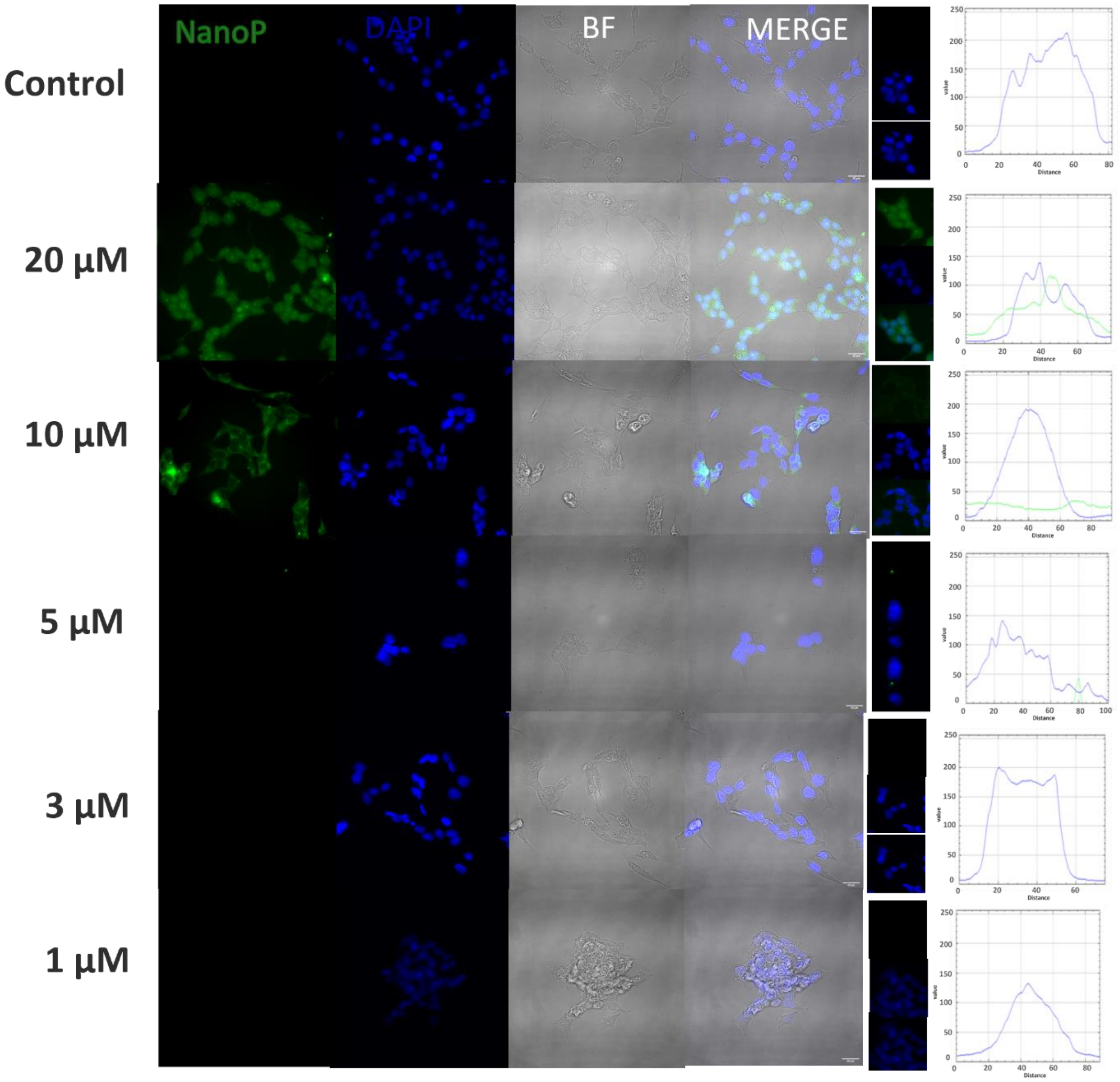

Supplementary Figure S2.4. Fluorescence microscopy of Curcumin nanoparticle in NCI-H295R cells. NCI-H295R cells were treated with increasing concentrations of Curcumin in the curcumin nanoparticle (20, 10, 5, 3, and 1 µM) for 4 hours. Cells were visualized using fluorescence microscopy to observe curcumin's cytosolic staining. **Scale bar**: 20 µm.

Supplementary Figure S3: Absorption test for the different concentrations (a) and the calibration curve of curcumin (b)

a

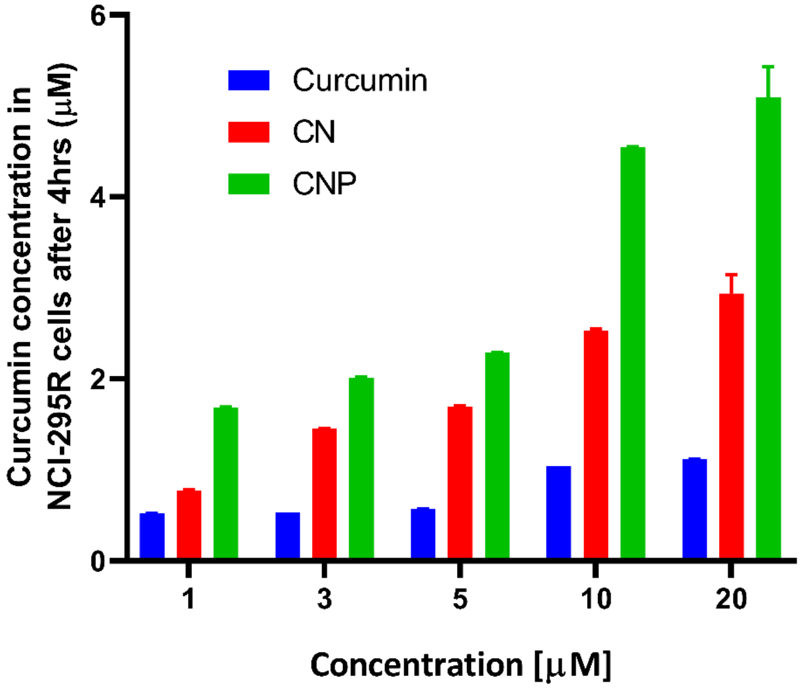

Supplementary Figure S3a: Absorption test for the different concentrations of curcumin

b

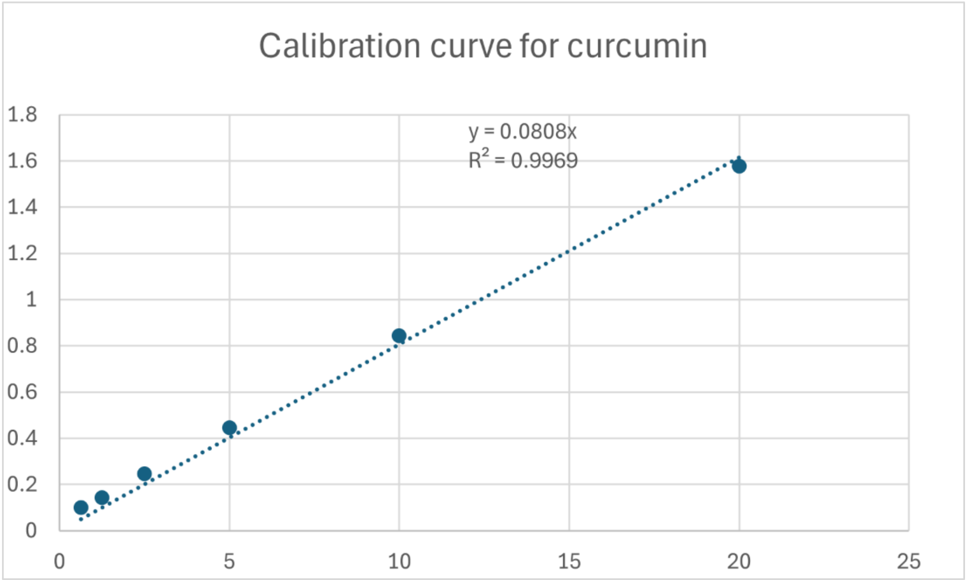

Supplementary Figure S3b: Calibration curve of curcumin

Supplementary Figure S4: Pictures of scratch assays.

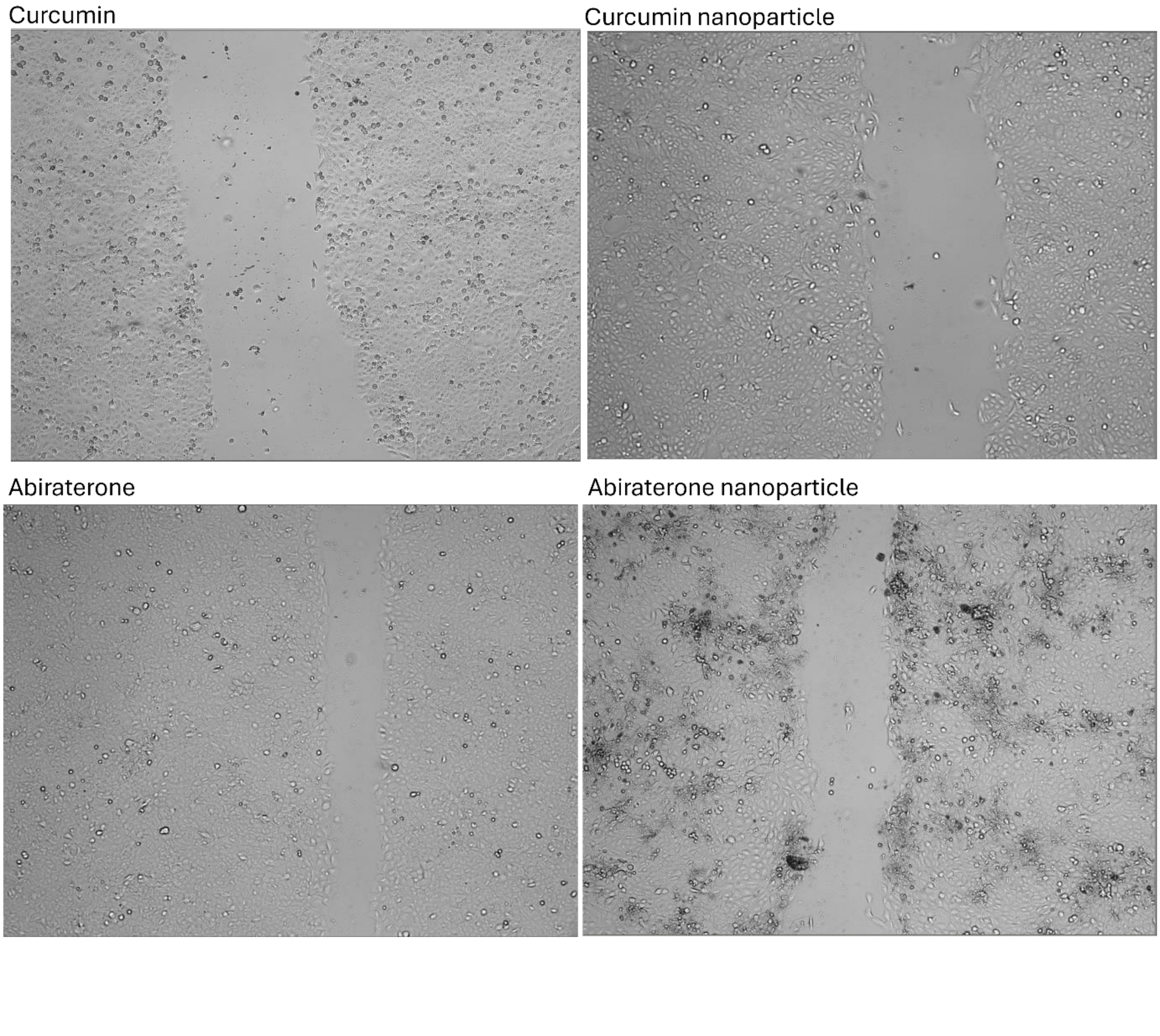

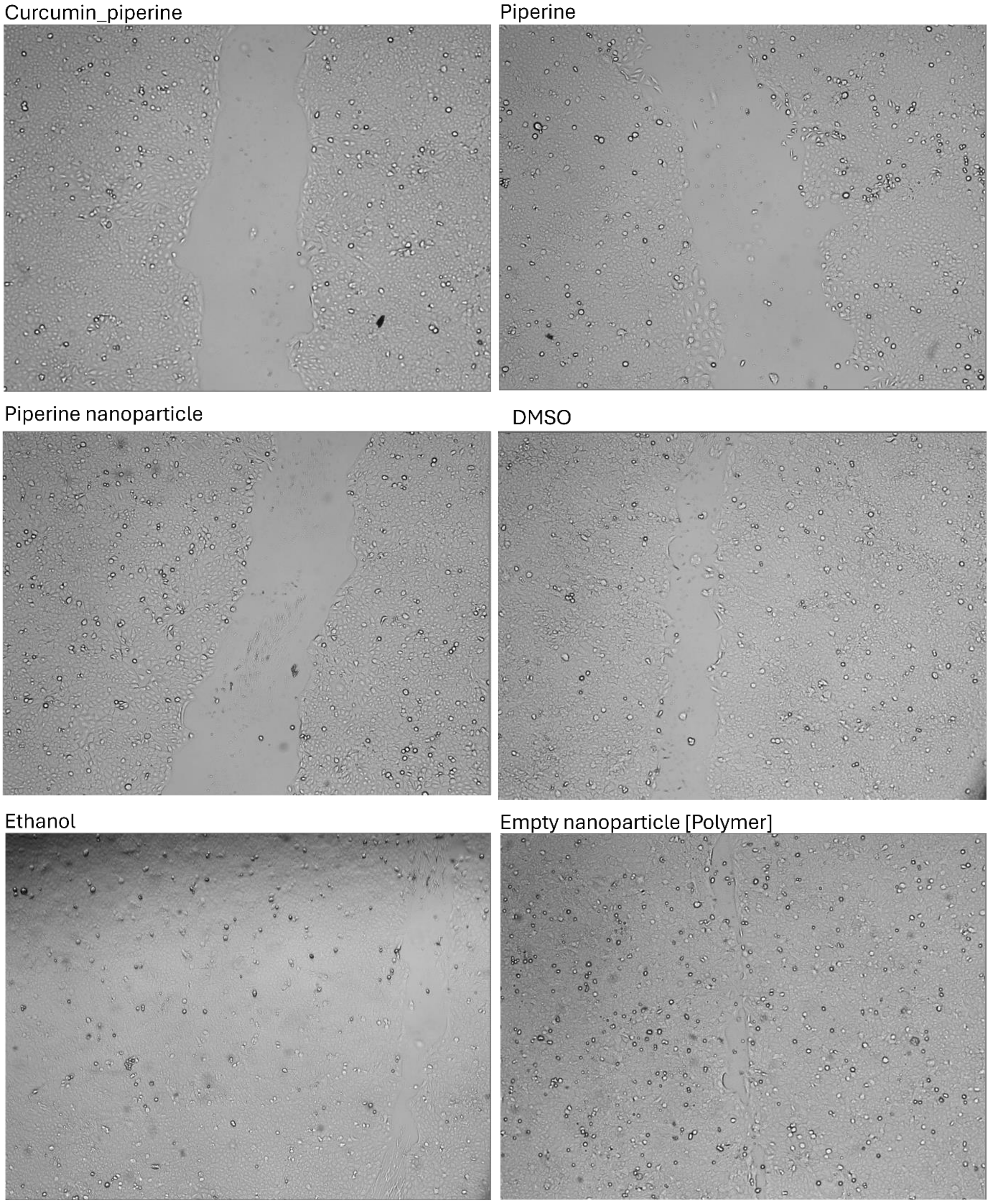

Supplementary Figure S4. Scratch assay images of DU-145cells treated with Curcumin, CN, and CPN. DU-145 cells were treated with Drugs at 10 µM for 24 hours. The images were captured using a bright field microscope after 24-hour post-treated. Representative bright field images showing the effect of drugs on cell migration.

Supplementary Figure S5: Cell cycle gated graphs and sample channel pics

Abiraterone_treated

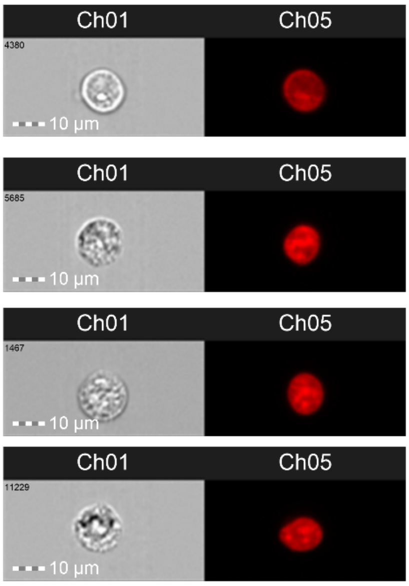

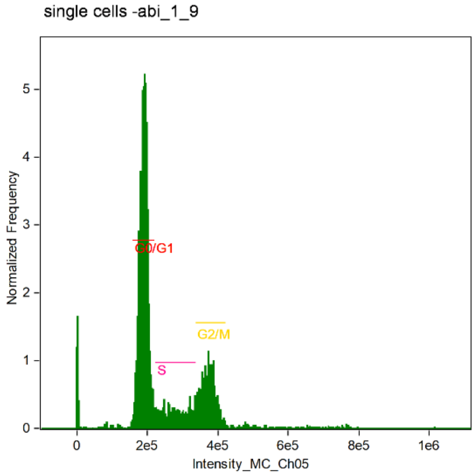

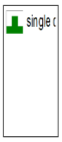

Intensity_MC_Ch05

Population	Count	%Total	%Gated
single cells & focus	4676	33.5	100
G2/M & single cells & focus	913	6.55	19.5
G0/G1 & single cells & focus	2895	20.8	61.9
S & single cells & focus	526	3.77	11.2

Abiraterone nanoparticle_treated

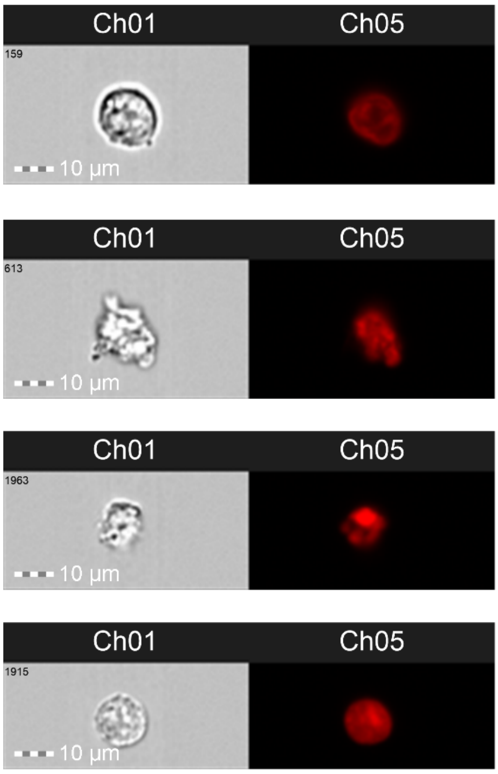

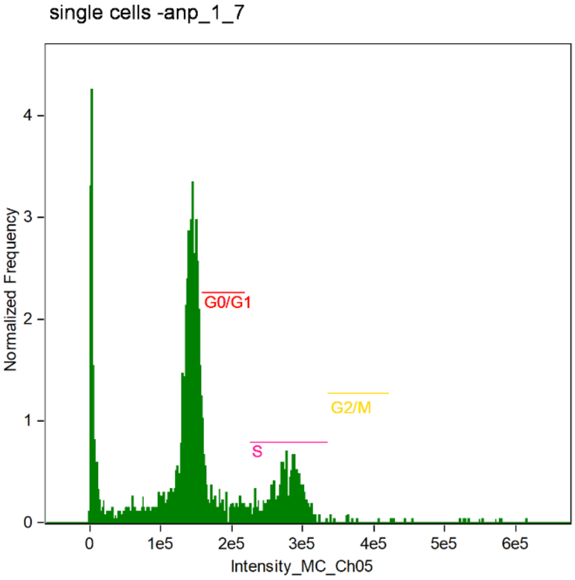

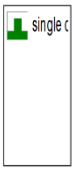

Intensity_MC_Ch05

Population	Count	%Total	%Gated
single cells & focus	2723	40.2	100
G2/M & single cells & focus	12	0.18	0.44
G0/G1 & single cells & focus	259	3.83	9.51
S & single cells & focus	473	6.99	17.4

Curcumin_treated

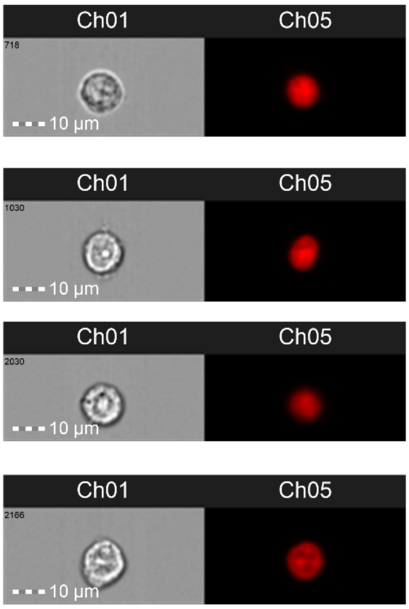

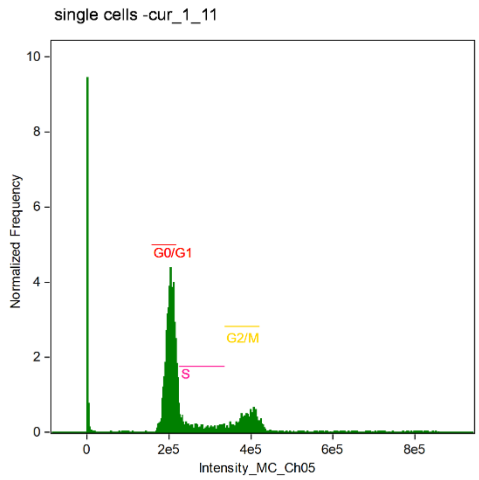

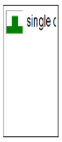

Intensity_MC_Ch05

Population	Count	%Total	%Gated
single cells & focus	4891	29.8	100
G2/M & single cells & focus	661	4.02	13.5
G0/G1 & single cells & focus	2576	15.7	52.7
S & single cells & focus	472	2.87	9.65

Curcumin nanoparticle_treated

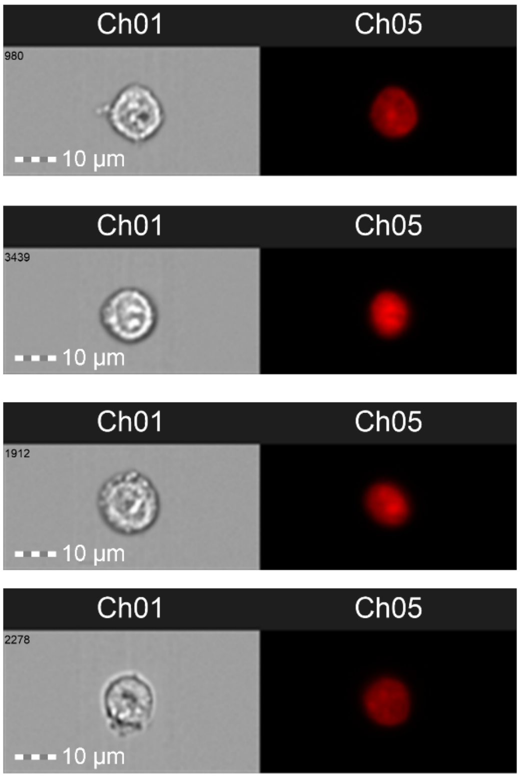

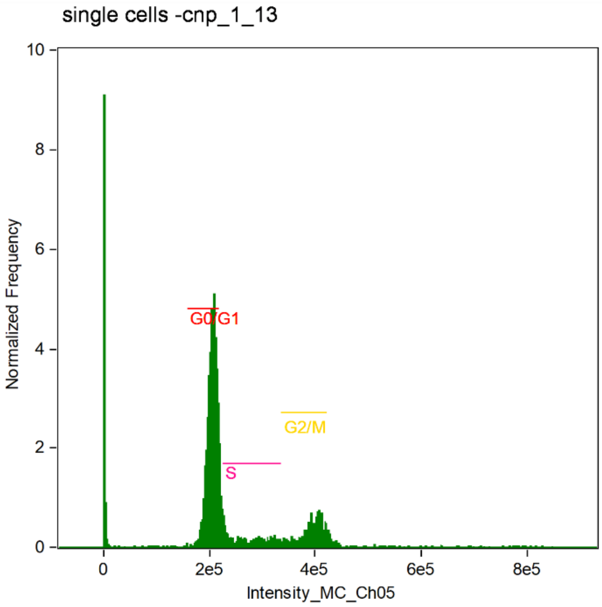

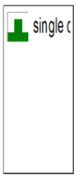

Intensity_MC_Ch05

Population	Count	%Total	%Gated
single cells & focus	4526	30.8	100
G2/M & single cells & focus	590	4.02	13
G0/G1 & single cells & focus	2412	16.4	53.3
S & single cells & focus	403	2.74	8.9

Curcumin_piperine_nanoparticle_treated

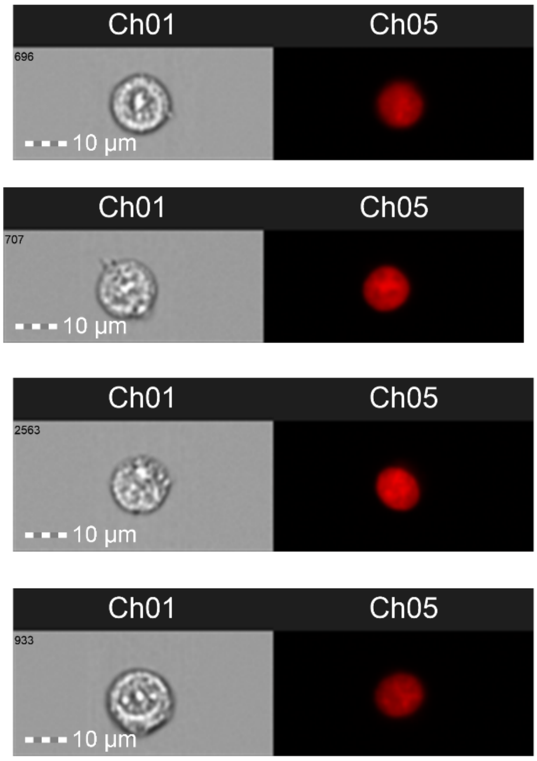

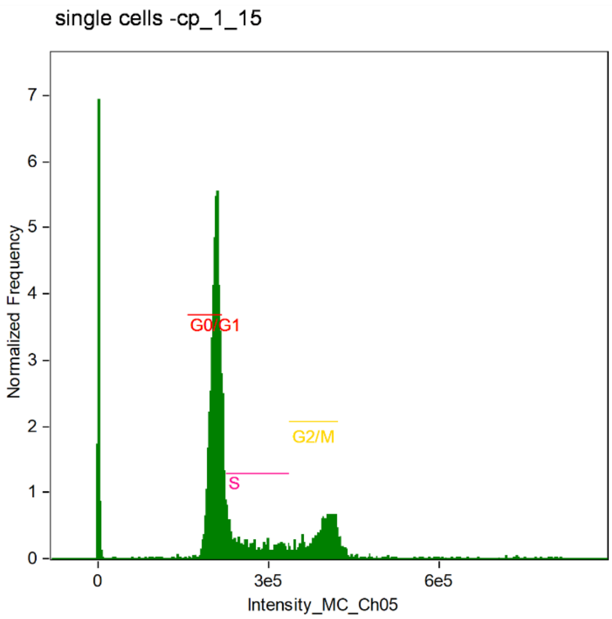

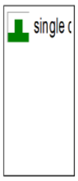

Intensity_MC_Ch05

Population	Count	%Total	%Gated
single cells & focus	5098	34.5	100
G2/M & single cells & focus	776	5.25	15.2
G0/G1 & single cells & focus	2783	18.8	54.6
S & single cells & focus	631	4.27	12.4

Piperine_treated

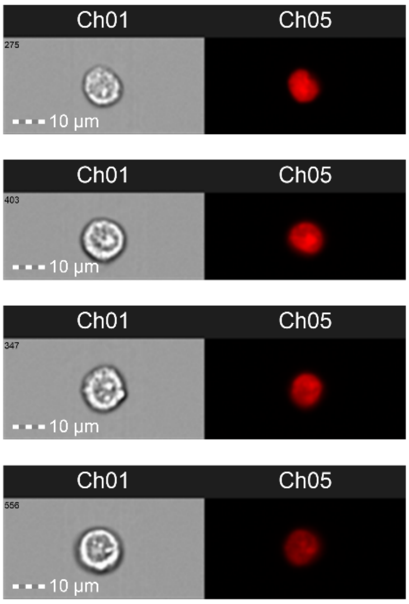

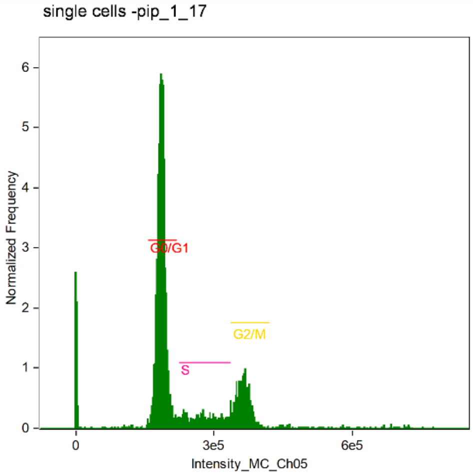

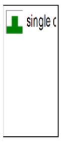

Intensity_MC_Ch05

Population	Count	%Total	%Gated
single cells & focus	4625	46.4	100
G2/M & single cells & focus	763	7.66	16.5
G0/G1 & single cells & focus	3002	30.1	64.9
S & single cells & focus	479	4.81	10.4

Piperine nanoparticle_treated

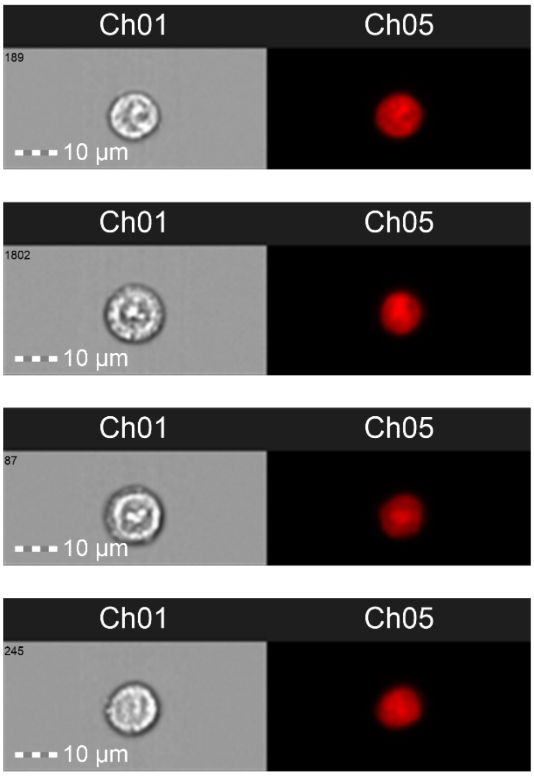

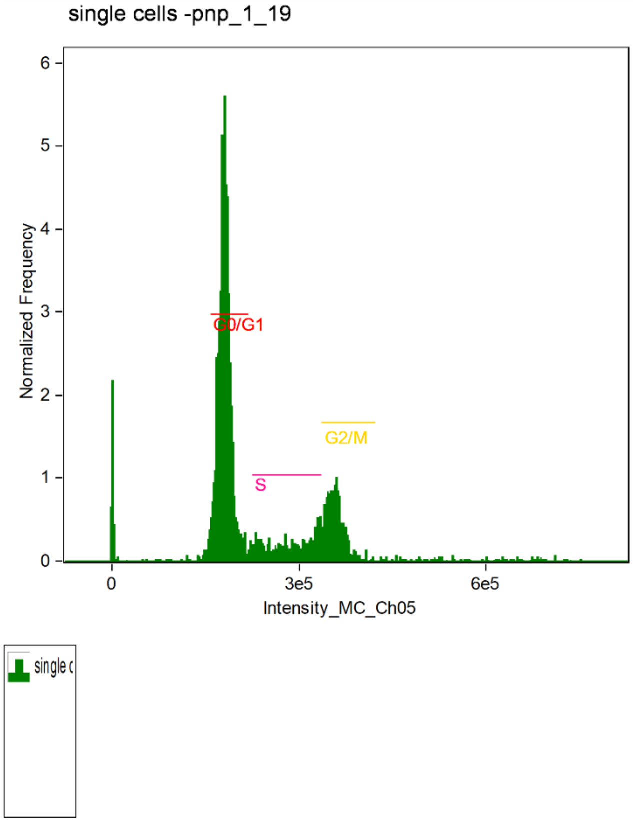

Intensity_MC_Ch05

Population	Count	%Total	%Gated
single cells & focus	4594	37.4	100
G2/M & single cells & focus	768	6.25	16.7
G0/G1 & single cells & focus	2872	23.4	62.5
S & single cells & focus	599	4.88	13

Dmso_treated

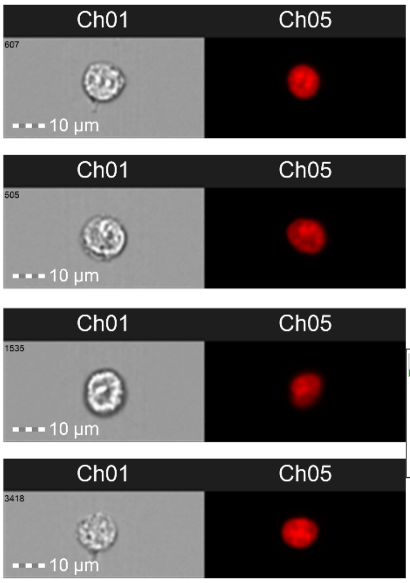

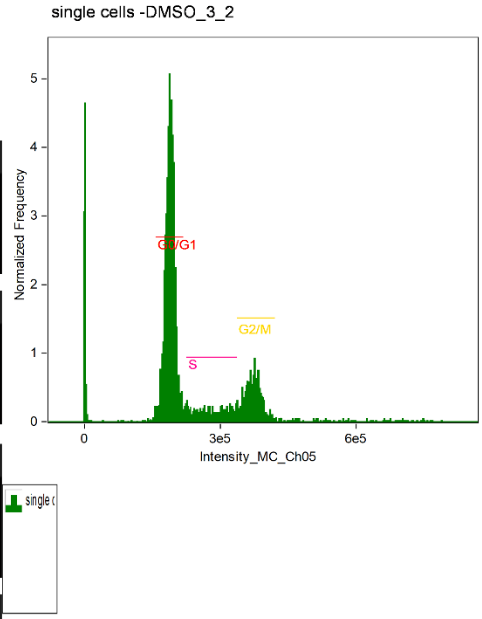

Intensity_MC_Ch05

Population	Count	%Total	%Gated
single cells & focus	4550	35.8	100
G2/M & single cells & focus	742	5.84	16.3
G0/G1 & single cells & focus	2838	22.4	62.4
S & single cells & focus	413	3.25	9.08

Empty nanoparticle [Polymer]_treated

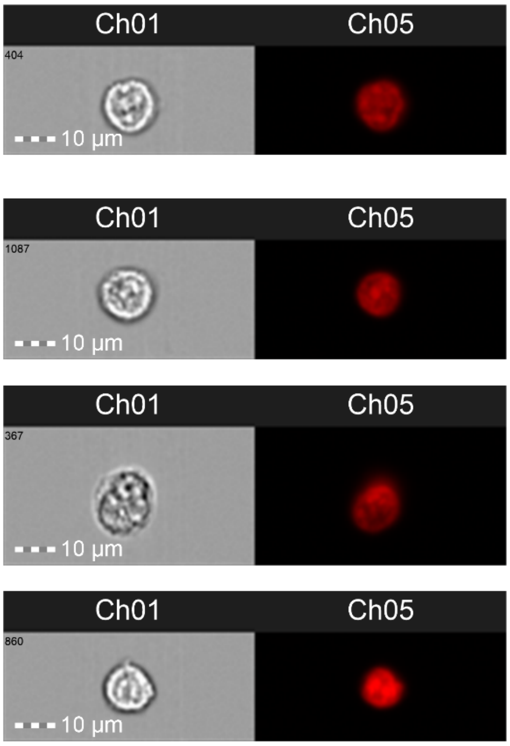

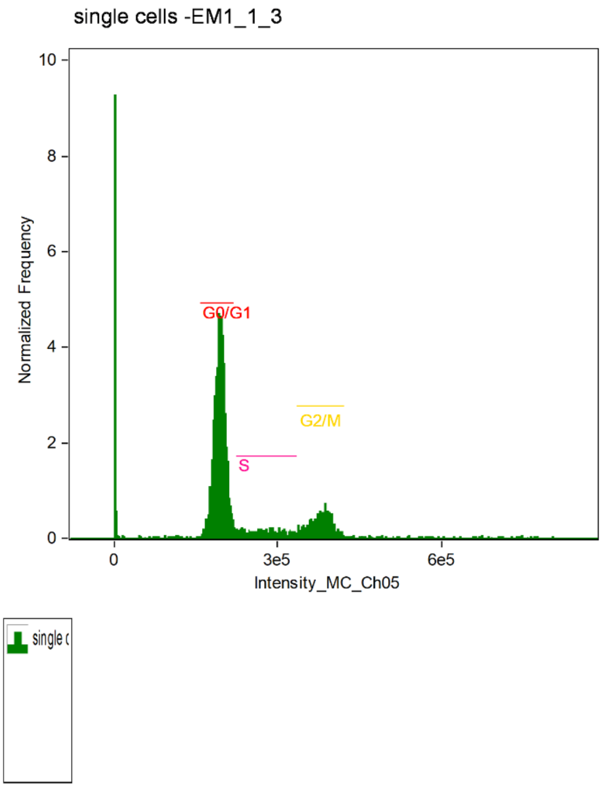

Intensity_MC_Ch05

Population	Count	%Total	%Gated
single cells & focus	4852	33.7	100
G2/M & single cells & focus	647	4.49	13.3
G0/G1 & single cells & focus	2900	20.1	59.8
S & single cells & focus	379	2.63	7.81

Ethanol_treated

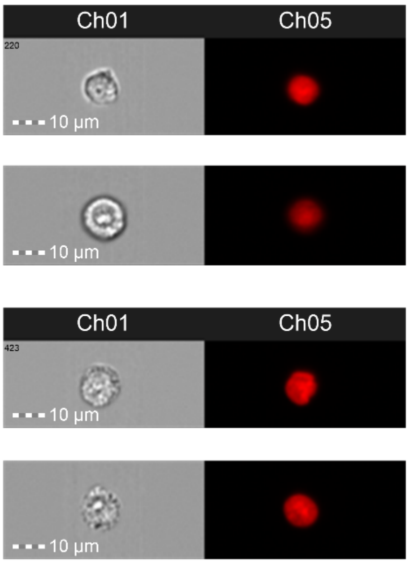

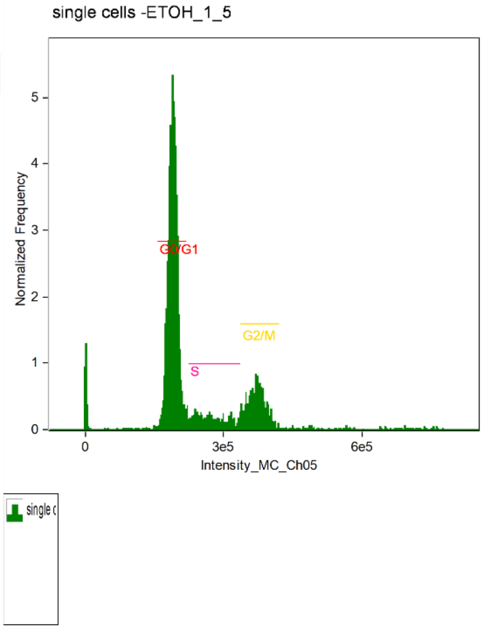

Intensity_MC_Ch05

Population	Count	%Total	%Gated
single cells & focus	4565	47.2	100
G2/M & single cells & focus	814	8.42	17.8
G0/G1 & single cells & focus	3033	31.4	66.4
S & single cells & focus	425	4.4	9.31

Supplementary Figure S5: The graphs depict the proportion of cells in the G0/G1, S, and G2/M phases, highlighting the changes in the cell cycle in response to the compounds.
